# Design and implementation of electronic health record integrated clinical prediction rules (iCPR): a randomized trial in diverse primary care settings

**DOI:** 10.1186/s13012-017-0567-y

**Published:** 2017-03-14

**Authors:** David A. Feldstein, Rachel Hess, Thomas McGinn, Rebecca G. Mishuris, Lauren McCullagh, Paul D. Smith, Michael Flynn, Joseph Palmisano, Gheorghe Doros, Devin Mann

**Affiliations:** 1 Division of General Internal Medicine, University of Wisconsin School of Medicine and Public Health, 2828 Marshall Court, Suite 100, Madison, WI 53705 USA; 2 Division of Health System Innovation and Research, University of Utah School of Medicine, Williams Building, 295 Chipeta Way, Salt Lake City, UT 84108 USA; 3 Department of Medicine, Hofstra Northwell School of Medicine, 300 Community Drive, Manhasset, NY 11030 USA; 4 Department of Medicine, Boston University School of Medicine, 801 Massachusetts Avenue, Crosstown 2, Boston, MA 02118 USA; 5 Department of Medicine, Hofstra Northwell School of Medicine, 600 Community Drive, Suite 300, Manhasset, NY 11030 USA; 6 Department of Family Medicine and Community Health, University of Wisconsin School of Medicine and Public Health, 1100 Delaplaine Court, Madison, WI 53715 USA; 7 Westridge Health Center, University of Utah School of Medicine, 3730 West 4700 South, West Valley City, UT 84118 USA; 8 Boston University School of Public Health, Fuller Building M-900C, Boston, MA 02118 USA; 9 Department of Biostatistics, Boston University School of Public Health, Crosstown Center-CT331, Boston, MA 02118 USA; 10 Department of Medicine, New York University School of Medicine, 227 East 30th St. 7th floor, New York, NY 10016 USA

**Keywords:** Clinical decision support, Electronic health record, Implementation science, Pneumonia, Pharyngitis, Randomized controlled trial, Streptococcal infections

## Abstract

**Background:**

Clinical prediction rules (CPRs) represent a method of determining individual patient risk to help providers make more accurate decisions at the point of care. Well-validated CPRs are underutilized but may decrease antibiotic overuse for acute respiratory infections. The integrated clinical prediction rules (iCPR) study builds on a previous single clinic study to integrate two CPRs into the electronic health record and assess their impact on practice. This article discusses study design and implementation of a multicenter cluster randomized control trial of the iCPR clinical decision support system, including the tool adaptation, usability testing, staff training, and implementation study to disseminate iCPR at multiple clinical sites across two health care systems.

**Methods:**

The iCPR tool is based on two well-validated CPRs, one for strep pharyngitis and one for pneumonia. The iCPR tool uses the reason for visit to trigger a risk calculator. Provider completion of the risk calculator provides a risk score, which is linked to an order set. Order sets guide evidence-based care and include progress note documentation, tests, prescription medications, and patient instructions. The iCPR tool was refined based on interviews with providers, medical assistants, and clinic managers, and two rounds of usability testing. “Near live” usability testing with simulated patients was used to ensure that iCPR fit into providers’ clinical workflows. Thirty-three Family Medicine and General Internal Medicine primary care clinics were recruited at two institutions. Clinics were randomized to academic detailing about strep pharyngitis and pneumonia diagnosis and treatment (control) or academic detailing plus use of the iCPR tool (intervention). The primary outcome is the difference in antibiotic prescribing rates between the intervention and control groups with secondary outcomes of difference in rapid strep and chest x-ray ordering. Use of the components of the iCPR will also be assessed.

**Discussion:**

The iCPR study uses a strong user-centered design and builds on the previous initial study, to assess whether CPRs integrated in the electronic health record can change provider behavior and improve evidence-based care in a broad range of primary care clinics.

**Trial registration:**

Clinicaltrials.gov (NCT02534987)

## Background

Patients receive only 55% of recommended care [1] along with a lot of unnecessary care [2]. While the amount of clinical evidence continues to explode, how to best integrate this evidence at the point of care remains elusive. In order to provide patients with the right care while avoiding unnecessary care, it is critical that we determine the best methods for making clinical evidence available to providers where clinical decisions are made.

Clinical prediction rules (CPRs) represent a method of determining individual patient risk to help decide what care is appropriate to give [3]. CPRs use data that can include patient history, physical exam findings, and basic lab test results to determine a patient’s risk for having a disease state. There are a number of well-validated CPRs that have been shown to be accurate and useful in reducing unnecessary care [4]. However, these CPRs are not being routinely used at the point of care and there are very few examples of integration into electronic health records (EHRs).

Overuse of antibiotics in respiratory tract infections has continued to be a major problem causing patient harm and contributing to antibiotic resistance [5–8]. We previously developed and validated an EHR-integrated clinical prediction rule (iCPR) clinical decision support (CDS) tool. The study demonstrated the tool’s ability to reduce inappropriate antibiotic prescribing and test ordering for patients with respiratory tract infections. However, it was developed and tested in a single academic health center internal medicine clinic, limiting its generalizability to more diverse settings [9]. To extend these promising findings, a new study was launched examining how these iCPR CDS tools translate to more diverse primary care settings. This article discusses the tool adaptation, usability testing, training, and implementation procedures used to adapt and disseminate the iCPR tool at diverse primary care clinics across two health care systems.

## Methods/design

The iCPR cluster randomized controlled study was designed to test the feasibility and effectiveness of incorporating strep pharyngitis and pneumonia CPRs into EHRs in diverse primary care practices. The main objective was to determine the impact of the iCPR on provider antibiotic prescribing and test ordering. This study was approved by each site’s Institutional Human Subjects Protection Review Board.

### Setting/clinic eligibility

The study is being conducted at primary care clinics associated with the University of Wisconsin and University of Utah medical centers. All General Internal Medicine (GIM) and Family Medicine (FM) primary care clinics at the two institutions were invited to participate. A total of 33 individual clinics (12 GIM clinics, 16 FM clinics, and 5 combined clinics) are participating in the study. Table 1 illustrates the clinic characteristics. Clinics were enrolled by site leads at each medical center. All physicians, nurse practitioners, physician assistants, and residents at participating clinics were eligible to participate. Both sites use the same EHR system (Epic Systems, Verona, WI) and had off-the-shelf capabilities to develop CDS tools in their EHR. Each site was supported by an information technology department that was able to develop and test the components of the iCPR before deployment.

**Table 1:** Description of study clinics

	University of Wisconsin	University of Utah
Total no. of clinics	22	11
No. of intervention clinics	12	6
Total no. of providers	268	111
GIM clinics	10	2
FM clinics	12	4
Combined GIM and FM clinics	0	5
No. of providers per clinic	2–29	3–23

*GIM* General Internal Medicine, *FM* Family Medicine

### Randomization

A computer-generated, blocked, stratified-randomization scheme was performed at the level of the clinic. Stratification was by institution and by the number of patient visits to the clinic in the previous year that would have triggered the iCPR tool. Three strata of visits were used: <750, 750–1500, and >1500. Group assignment was performed by the study statistician.

### Intervention and control groups

Both groups received a 45-min academic detailing session that included a review of the CPRs used in the study, discussion of evidence-based diagnosis of strep pharyngitis and pneumonia, and guidelines for treating strep pharyngitis and pneumonia. Participants were given handouts with the CPRs and treatment guidelines and links to online resources. Academic detailing sessions at intervention clinics also included an overview of the iCPR tool with a live demonstration in the EHR. Participants received handouts about iCPR and links to additional online training materials. Providers that were unable to attend the academic detailing were given access to printed and online training materials. The iCPR tools were made active in the EHR for providers at intervention clinics on the day of the academic detailing, thus giving providers immediate access. Control groups did not receive access to the iCPR tools.

### Patient inclusion and exclusion criteria

Patients are included in analyses if they have a visit with a provider at a study clinic during the study period that meets iCPR triggering criteria based on reason for visit, diagnosis, or diagnosis and antibiotic ordering (Table 2). In addition, patients must meet the age criteria for tool use: ages 3 to 70 years for possible strep and ages 18 to 70 for possible pneumonia. Age cutoffs were based on the validation evidence for the CPRs [10–12]. While validation studies did not necessarily have an upper age cutoff, few patients older than 70 were included in these studies and presentation of respiratory infection may change with age. A waiver of informed consent was obtained from the Institutional Human Subjects Protection Review Board at each medical center.

**Table 2:** iCPR triggers (reason for visit, diagnosis, and combined diagnosis/antibiotic)

Strep	Pneumonia	Both
Reason for visit		
Sore throat	Cough and/or chest congestion	
	URI symptoms	
Diagnosis		
Pharyngitis (ICD-10: J02.9, R07.0)	Cough (ICD-10: RO5)	
Strep (ICD-10: J02.0, J03.00, J03.01)	URI (ICD-10: J22, J98.8, J06.9)	
	Bronchitis (ICD-10: J20.8, J20.9, J40)	
	Pneumonia (ICD-10: J13, J18.1, J15.0, J14, J15.4, J15.3, J15.20, J15.211, J15.212, J15.29, J15.5, J15.6, J15.8, J15.9, A48.1, J18.9)	
Reason for visit and antibiotic combination^a^		
Hoarseness	Fever	
Diagnosis and antibiotic combination^a^		
Laryngitis (ICD-10: J04.0, J04.2, J05.0, J06.0)	Wheezing (ICD-10: R06.2)	Dyspnea/SOB (ICD-10: R06.89, R06.09, R06.00, R06.02)
		Fever (ICD-10: R50.9)
		Rhinitis (ICD-10: J00)

*URI* upper respiratory infection, *ICD* International Classification of Diseases, *SOB* shortness of breath

^a^Antibiotics: Oral penicillins, macrolides, cephalosporins, quinolones, tetracyclines

### Tool adaptation

The tool adaptation process consisted of several steps to ensure it satisfied the variable workflows and clinical content needs of each site.

#### Clinical prediction rules

We focused on respiratory tract infections and chose well-validated CPRs for evaluating the risk of streptococcal pharyngitis (sore throat) and pneumonia (cough). We chose the Centor criteria [10] for adults with sore throat which includes four criteria: absence of cough, pharyngeal exudates, tender anterior cervical lymphadenopathy, and fever. We chose the McIssac criteria [13] for children with sore throat which mirrors Centor criteria with the addition of patient age. We chose the Heckerling criteria [12] for adults with risk of pneumonia which include five criteria: fever, increased heart rate, crackles, decreased breath sounds, and absence of asthma (Table 3).

**Table 3:** Clinical prediction rules for strep pharyngitis and pneumonia

Strep pharyngitis			Pneumonia
	Children	Adults	Adults
Age range	3–17 years old	18–70 years old	18–70 years old
Rule	McIsaac [13]	Centor [10]	Heckerling [12]
Criteria	Tonsillar exudate +1 Tender anterior cervical adenopathy +1 Lack of cough +1 History of fever +1 3–14 years old +1	Tonsillar exudate +1 Tender anterior cervical adenopathy +1 Lack of cough +1 History of fever +1	Temperature > 100 F +1 HR > 100 bpm +1 Crackles (rales) +1 Decreased breath sounds +1 Absence of asthma +1

*HR* heart rate, *bpm* beats per minute

#### Comparing workflows

The current iCPR tool is adapted from the previous iCPR tool but tailored to fit the current sites’ unique workflows. The iCPR tool design was developed by an interdisciplinary team of experts in primary care, usability, and clinical informatics. Interviews were held with providers, clinic managers, and medical assistants at each site to determine general clinic workflows as well as specific workflows for rapid strep and chest x-ray testing.

These interviews demonstrated that workflows varied dramatically by institution with some variation by clinic and even by provider within a clinic. For example, a major difference between institutions was that University of Utah providers heavily leveraged an EHR-assisted documentation pathway called “NoteWriter.” This structured documentation template has the ability to create pick-list histories based on the patient’s chief complaint. In this workflow, medical assistants record a structured history that the provider then reviews and confirms with the patient. The structured history, as well as the vital signs and, if used, structured physical exam then populates the iCPR tool. This decreases the need for duplicate documentation. Another workflow difference was a variable approach among clinics to rapid strep testing including which clinic personnel review test results and whether patients remain in the clinic until results are complete.

#### Updating clinical content

The clinical content of the iCPR order sets and the underlying triggering logic required review and revision to meet current national and local standards of care. An interdisciplinary team of experts in primary care, infectious disease, laboratory medicine, and clinical informatics met to determine appropriate medical care for patients with sore throat and cough for varying disease risk levels. National guideline recommendations, clinical studies, and local antibiotic resistance and practice patterns guided the group’s choices regarding the iCPR tool content. For example, first-line antibiotic choices for pneumonia were based on the Infectious Disease Society of America’s guideline [14] but tailored to local strep pneumonia resistance patterns.

### Tool design considerations

The team also reviewed all of the iCPR tool features and designs to ensure it met the needs of the diverse clinics. This included examining the (1) tool activation level, (2) timing of alerts, (3) integration into clinical workflow, (4) alert triggers, and (5) interruptive versus non-interruptive alerts.

1. Tool activation: We chose to activate the tool based on the clinic where the encounter was performed instead of at the provider level. The clinic was chosen to coincide with the unit of randomization and to prevent study contamination from providers that worked at multiple clinics.
2. Timing of alerts: It was clear from interviews with providers that there was variation in when they ordered tests and antibiotics during the patient encounter. We chose to base the main trigger on the reason for visit despite infrequent completion of this field by providers in the original iCPR clinical site [9]. Relying on reason for visit as the main trigger made iCPR available early in the encounter and throughout the subsequent workflow. We also included a secondary trigger, similar to the original iCPR design, that would occur toward the end of the encounter based on diagnosis and a combination of diagnosis and orders. While the secondary trigger may not be frequently used, it might allow providers to change decisions or at least alert them to considerations for future encounters. We also automatically triggered alerts in the provider’s inbox and when the provider reopened an encounter after a relevent test had resulted.
3. Integration into clinical workflow: The iCPR needed to be integrated into active clinical practice without disrupting patient care. It is clear that without integration into workflow, CDS tools are not used [15]. This led to some individualization between institutions including the use of NoteWriter at some sites. We also decided to make iCPR as flexible as possible in order to facilitate the integration into various workflows. This included the ability for providers and nurses to use bundled ordersets based on test results and for support of telephone encounters or patient portal encounters.
4. Alert triggers: Another guiding principle was limiting the number of inappropriate triggers. We balanced trigger sensitivity and specificity, erring on the side of specificity at the cost of missing some potentially appropriate encounters. Triggers were determined by reviewing clinic data from the previous year for the final encounter diagnoses based on available reasons for visit and whether antibiotics were prescribed. We found that only three reasons for visit commonly resulted in diagnoses that we wanted to affect. Based on these, we chose “cough/chest congestion,” “URI (upper respiratory infection),” and “sore throat” as the reason for visit triggers. For triggers based on diagnosis and antibiotics, we chose the narrowest diagnoses and only antibiotics commonly used in respiratory infections (Table 2).
5. Interruptive vs non-interruptive alerts: Interruptive alerts interfere with workflow and force users to acknowledge the alert, potentially increasing clinician frustration and alert fatigue [16, 17]. Given the broad nature of the three reasons for visit triggers we chose, we knew that iCPR was likely to trigger at times when it would not be helpful, such as when a patient with asthma or sinusitis presented with a cough. Based on the risk of inappropriate triggers as well as provider opinions from the interviews, we chose to use non-interruptive alerts for the reason for visit triggers. We explored options for non-interruptive alerts for the diagnosis triggers, but there were no mechanisms within the EHR that allowed us to do this. Thus, we opted for interruptive alerts if iCPR was triggered by a diagnosis or diagnosis plus antibiotic prescription.

### iCPR components

**Fig. 1:**
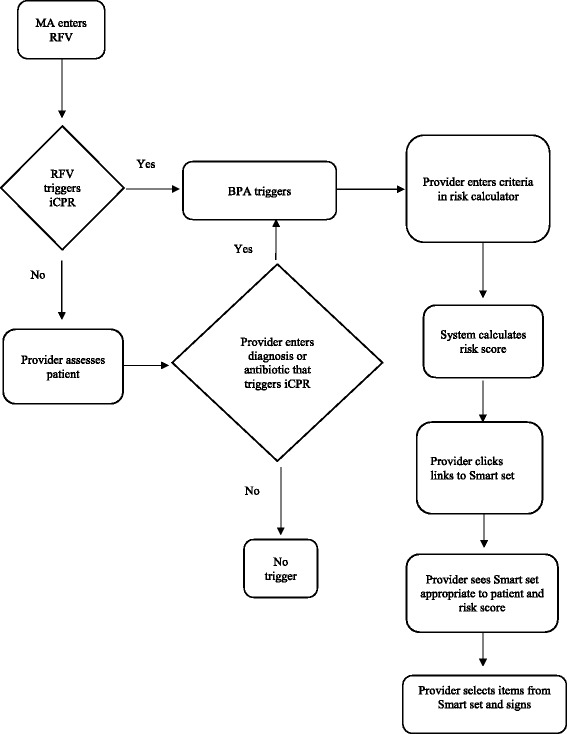
iCPR work flow. *MA* medical assistant, *RFV* reason for visit, *BPA* best practice alert

iCPR is triggered when a matching reason for visit, diagnosis, or diagnosis plus antibiotic order is entered (Table 2). This initiates an alert to the provider that decision making regarding diagnostic and treatment options for the patient might be assisted by the use of iCPR. Providers can then choose to click on a link and go to the risk calculator. Once the risk calculator is completed, another alert provides a link to access bundled order sets that include orders, documentation, diagnosis, and patient education materials (Fig. 1).

#### Alerts

At the University of Wisconsin, a standard EPIC alert is used to inform providers when a patient is appropriate for iCPR. Providers are familiar with seeing these alerts for other conditions. The alert specifies why iCPR was triggered and includes a link to the risk calculator. In addition to this alert, the University of Utah placed an additional tab in NoteWriter. This tab, called “Provider Score,” was populated with the appropriate iCPR criteria (Centor, McIsaac, and/or Heckerling) based on the reason for visit. In all other cases, it was blank. The Provider Score tab of NoteWriter draws information from vital signs and structured history and physical exam documentation. It can also be completed ad hoc. After completion, the score and interpretation are visible to the provider. The calculator score also drops directly into the clinical note.

#### Risk calculators

**Fig. 2:**
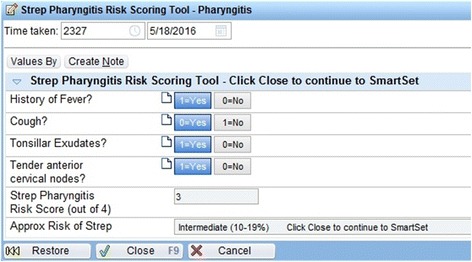
iCPR risk calculator example. © 2017 Epic Systems Corporation. Used with permission

We chose documentation flow sheets for the calculator to allow calculation of risk scores based on CPR criteria (Fig. 2). All calculators use simple yes/no buttons for choosing if the criteria were met. The age in the McIssac criteria is automatically entered from the birthdate in the EHR, and the heart rate and temperature are automatically entered in the Heckerling criteria from the current encounter vitals. Calculators display a risk score from 1–5 and the range (low, intermediate and high) of risk of strep pharyngitis or pneumonia based on results from previous validity studies [18, 19].

#### Smart sets

**Fig. 3:**
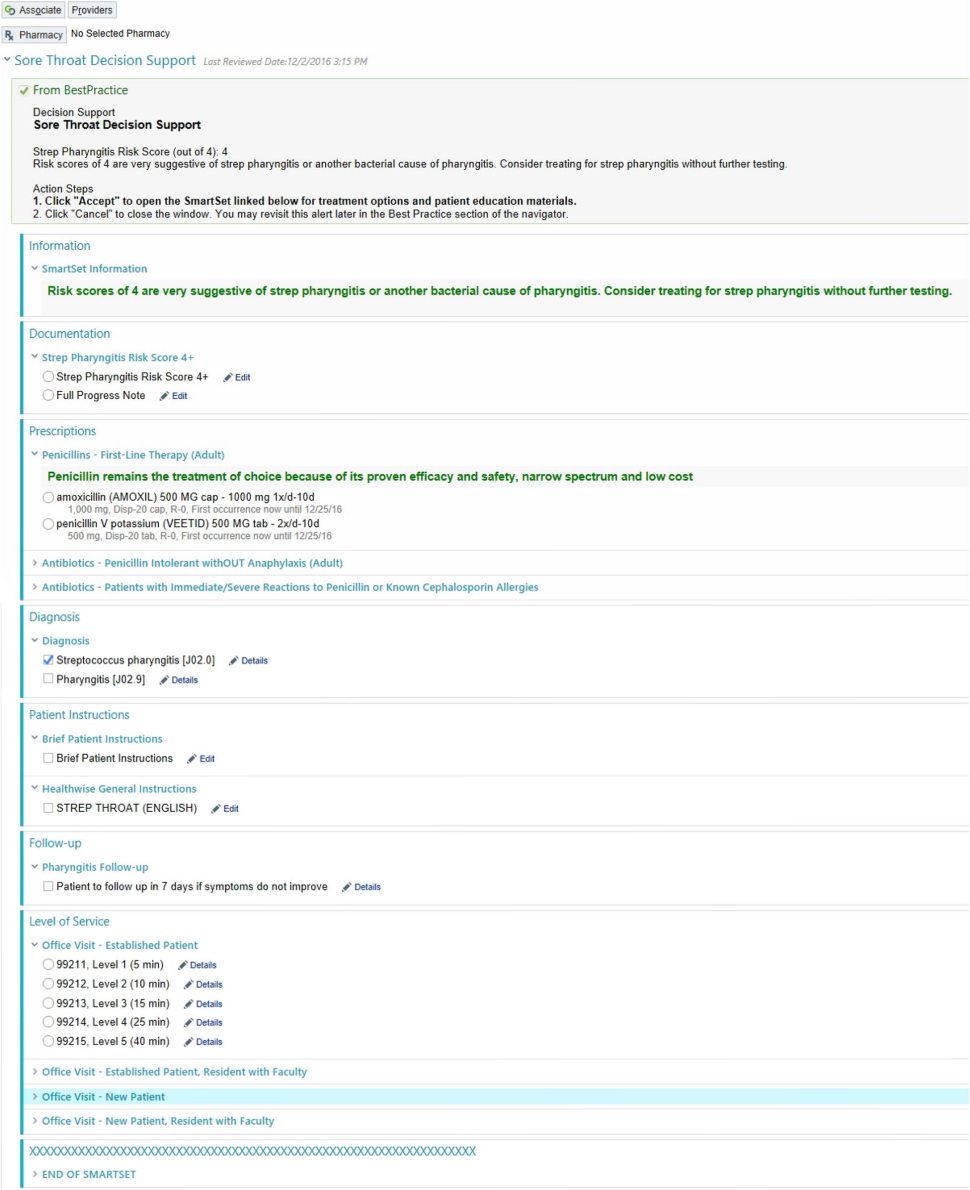
iCPR smart set example. © 2017 Epic Systems Corporation. Used with permission

The content of the smart sets needed to vary by the level of patient risk (low, intermediate, or high). To provide this functionality, we chose to leverage EPIC functionality which allowed suppressing components of the smart set based on patient factors (weight and age) as well as risk score. We developed one smart set for sore throat and one for cough. The smart sets included documentation for progress notes, laboratory orders, prescription orders, diagnoses, patient instructions, and level of service (Fig. 3).

### Usability testing

#### “Think aloud”

Think aloud testing was performed to determine the usability of the individual iCPR components. Primary care providers, six from the University of Utah and four from the University of Wisconsin, were selected from volunteers to form a convenience sample. Inclusion criteria required that participants worked in Family Medicine, Internal Medicine, or Urgent Care clinics, spent at least 50% of their time providing clinical care, and were currently using the EHR system in which the CDS would be imbedded. Each participant was presented with a written clinical case describing a patient with low, intermediate, or high risk of either strep pharyngitis or pneumonia. Under scripted instruction from the interviewer, the participant was directed to perform different aspects of clinical documentation including opening the chart, entering patient data, creating a progress note, and placing appropriate orders. While interacting with the tool, participants were strongly encouraged to think out loud and to verbalize their thought process. After interacting with the tool, the participant was asked a few specific questions about general attitudes toward the tool. Each session lasted 25–45 min. Screen capture and audio were recorded and put into categories by two independent coders.

#### “Near live”

The goal of the near live testing was to determine how the tool fits with providers’ workflows. Eight primary care providers from the University of Wisconsin were selected from volunteers. Inclusion criteria were similar to those used in the think aloud testing. In a clinic office setting, each participant interacted with a simulated patient, an actor who was trained to portray a case of low-, intermediate-, or high-risk strep pharyngitis or pneumonia. The participant interacted with the patient actor while navigating through the CDS tool. Participants were told to think out loud if they had any challenges or positive experiences with the tool. The study staff observed the sessions to answer questions, troubleshoot the software, and provide the physical exam information. After completing the testing, participants were asked about their reaction to specific components of the tool and for suggestions for improvement. Every participant completed at least one case, and two completed two cases each. The duration of each session was between 25–45 min. Two independent coders reviewed all recorded screen captures and the transcribed audio. Verbalized thoughts and participant actions were coded. Based on usability testing, multiple modifications were made to the wording and format of the alerts, calculators, and smart sets. Also, specific ranges of risk were included in the calculator and some non-antibiotic medication orders were removed from the smart sets.

### Outcomes

#### Evaluation framework

RE-AIM is a five-part framework designed to enhance the quality, speed, and public health impact of efforts to translate research into practice [20]. The five dimensions of RE-AIM are reach, effectiveness or efficacy, adoption, implementation, and maintenance. The framework has guided successful implementation and dissemination projects across disease entities and health care settings [21–23]. It encourages a study that balances concern for internal and external validity, giving equal attention to efficacy and to generalizability and dissemination potential [24]. Our evaluation plan will incorporate all five dimensions of the RE-AIM framework. REACH: To ensure that we reach the targeted audience, we will evaluate the percent of primary care clinics at each institution that participate in the study and compare the specialty, size, and location of participating versus non-participating clinics. EFFICACY: To determine efficacy of the ICPR tool, we will evaluate the clinical practice outcomes discussed below. ADOPTION: To evaluate adoption of the iCPR tool, we will determine provider utilization by clinic and institution as discussed in the process outcomes section below. IMPLEMENTATION: Fidelity of iCPR tool delivery will be evaluated via the number of attendees at academic detailing sessions and use of all components of the tool. MAINTENANCE: Comparison of adoption and efficacy trends from year one to year two will be used to determine whether the tool has become routine practice at the two institutions.

#### Clinical data collection

All clinical data will be collected via the EHR. Provider data will include level of training (physician, resident physician, NP, PA), gender, and date of birth. Provider data is stored in the provider profile within the EHR. Patient data will be collected for patients that have iCPR eligible encounters during the study period. Patient data will include date of birth, allergies, comorbidities, and any other encounters within 1 week of the qualifying encounter. Encounter data will include clinic site, date of visit, medication orders (including antibiotics), test orders (rapid strep test, throat culture, and chest x-ray), test results, diagnoses, trigger for iCPR (reason for visit or diagnosis), use of iCPR components, and score on iCPR calculator. Collection of data in the control group will use a “shadow” simulation to determine which encounters meet criteria for iCPR trigger even though the tool does not actually trigger. The final data set will be shared with the principal investigator, co-investigators, and the data-coordinating site at Boston University. The funding agency NIAID will also have access to use the data set.

#### Clinical practice outcomes

The study outcomes were designed to evaluate changes in clinical practice related to iCPR tool use. The primary outcome is the difference in antibiotic prescribing rates in iCPR patient encounters in intervention versus control providers. Secondary outcomes include the rates of rapid strep, throat culture, and chest x-ray ordering and the class of antibiotics prescribed. To measure the safety of clinical care, we will evaluate rates of additional encounters to primary care or urgent care, hospitalization, and prescription of respiratory tract antibiotics within 1 week of the index encounter.

#### Process outcomes/iCPR implementation

Success of clinical decision support relies on uptake of the tools into practice. It is critical to determine how the tool is being used in practice in order to understand why clinical practice outcomes changed. The process measures will help determine iCPR use in general as well as the use of specific components of the tool. Process outcomes will include completion of the risk calculator and use of the individual components of the smart sets. We will use Normalization Process Theory (NPT) to better understand the factors that affected implementation in individual clinics. NPT provides a framework to evaluate organizational impact as well as facilitators and barriers to implementation [25]. The 16-item NPT questionnaire contains questions in four domains: sense-making, participation, action, and monitoring. Questionnaires will be completed by intervention clinic medical directors and managers at baseline and every 6 months until completion of the 2-year study. Changes over time will be evaluated and qualitative assessment will be done for differences between clinics with high and low adoption.

### Data monitoring

Weekly reports are generated to track the frequency of tool triggering, calculator completion, and smart set usage by clinic. This will allow us to determine if iCPR is being used and review trigger rates at each clinic to evaluate issues with triggering. If provider use of iCPR is lower than expected, we will contact clinic medical directors and managers to determine potential issues with the tool. We will also perform intermittent chart review to ensure that iCPR is triggering properly and data collection is correct. Modifications to iCPR to improve usage or data collection may be made based on the findings of the data monitoring. A data monitoring committee is not required since there is minimal risk of harm from using the CDS that supports guideline-based care.

### Statistical analysis

Planned statistical analyses include comparison of patient and provider characteristics between groups to evaluate for group balance. Descriptive statistics will be used to show the use of iCPR components in the intervention group. A three-level logistic regression model with a random effect for practice and one for provider within practice will be used to assess the primary outcome and other clinical outcomes. A fixed effect will be included for intervention and for randomization strata. Subgroup analysis by primary institution (Wisconsin or Utah) and provider training will be performed. An interim analysis will be done 1 year after the last clinic receives academic detailing. Comparison of iCPR component use based on provider training and gender will be performed using a three-level logistic regression model with a random effect for practice and for provider within practice with fixed effects for provider training and gender.

### Power calculation

Sample size calculations were adjusted for clustering of patients within clinics. Prior year visit data from the University of Wisconsin and the University of Utah was used to estimate the number of iCPR triggers during the study period. We assumed 25 clusters or clinics with a control group antibiotic prescribing rate of 30–40% for iCPR eligible encounters and an absolute decrease of 10% in the rate of antibiotic prescribing in the intervention group. The intra-cluster coefficient was estimated to be between 0.01 and 0.05. Sample size calculations were performed with a significance level of 0.05 and 80% power. The estimated sample size for the most conservative assumptions (control group antibiotic prescription rate of 40% and intra-cluster coefficient of 0.05) is 52,457 patient encounters which, based on historical data, should be attained within 2 years.

### Implementation

**Fig. 4:**
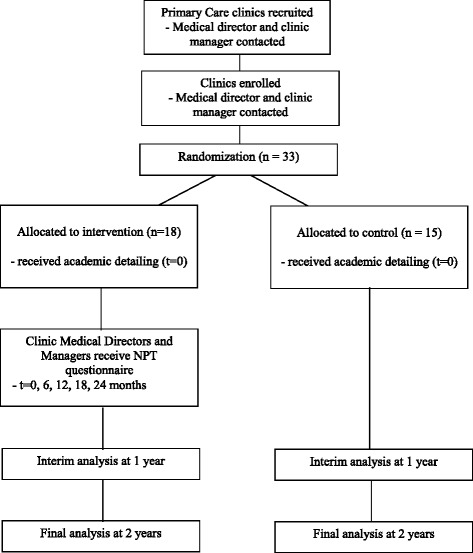
iCPR study flow. *NPT* normalization process theory

We used a staggered roll out of iCPR to evaluate for any problems, ensure appropriate training, and make sure that there was no interference with patient care. iCPR was first deployed at one GIM and one FM clinic. Researchers followed up with the clinic manager and medical director at these clinics within 2 weeks to determine if the rollout should continue. No major problems were identified, and the rollout continued to the remainder of the clinics over the next 12 weeks based on clinic availability for academic detailing. Following implementation in the remainder of the clinics, managers and medical directors were again contacted to determine if any problems had arisen. A contact link was also built into the iCPR alert so that individual providers could contact the researchers with any questions or concerns. The study was launched in October 2015 and is ongoing (Fig. 4).

### Protocol amendments

Changes to the research protocol that impact conduct of the study will be reviewed by the individual institutional review boards. Ammendments will also be made to the trial registry as necessary.

### Confidentiality

Participants’ anonymity will be maintained. Depersonalized data will be extracted from the EHR and stored on secure servers at each medical center. Transmission of data for analysis will occur via secure file transfer protocol (s-ftp). All documents will be stored securely and accessible only by the trial investigators.

### Dissemination

Trial results will be published in an open access medical journal and posted on ClinicalTrial.gov.

## Discussion

The multi-site iCPR trial builds on the previous single-site study to assess whether CPRs integrated in the EHR can change provider behavior and improve evidence-based care in a broad range of primary care clinics. We focused on the importance of integrating the tool into clinic workflows in order to optimize uptake and clinical usefulness. This required building flexibility into the tool to accommodate a variety of workflows and led to variations in the tool design and workflows between the two health systems. Near-live usability testing evaluated how well we succeeded in workflow integration and reaffirmed the importance of near-live usability testing when implementing new EHR tools. While we designed the tool to be provider-centric, we were forced to make some usability compromises based on limitations in the EHR. The limited points for iCPR triggering and lack of specificity of potential triggers were highlighted in usability testing. However, there were no clear solutions to these issues within the current EHR. EHR systems need to continue to evolve in order to better accommodate the diversity in workflow and clinical decision making. The impact of iCPR on antibiotic prescribing remains to be determined; however, our experience developing this tool for a diverse group of clinics and having clinicians and clinic personnel involved throughout the development process represent a roadmap for delivering evidence-based tools through CDS at the point of care.

## Abbreviations

BPM: Beats per minute
CDS: Clinical decision support
CPR: Clinical prediction rule
EHR: Electronic health record
FM: Family Medicine
GIM: General Internal Medicine
HR: Heart rate
iCPR: Integrated clinical prediction rules
NPT: Normalization Process Theory
URI: Upper respiratory infection
s-ftp: Secure file transfer protocol
